# Microvascular effects of a mixed meal tolerance test: a model validation study

**DOI:** 10.1111/cpf.12904

**Published:** 2024-09-23

**Authors:** Sebastiaan J. W. van Kraaij, Boukje Charlotte Eveleens Maarse, Femke P. M. Hoevenaars, Ines Warnke, Marieke L. de Kam, Matthijs Moerland, Pim Gal

**Affiliations:** ^1^ Centre for Human Drug Research Leiden The Netherlands; ^2^ Leiden University Medical Centre Leiden The Netherlands; ^3^ TNO, Netherlands Organisation for Applied Scientific Research Leiden The Netherlands; ^4^ dsm‐firmenich CH‐4303 Kaiseraugst Switzerland

**Keywords:** endothelial function, laser speckle contrast imaging, passive leg movement, sidestream dark field microscopy

## Abstract

**Purpose:**

Endothelial dysfunction is a pathophysiological change preceding many cardiovascular events. Measuring improvements of endothelial function is challenging when function is already optimal, which may be remediated using a physiological challenge. This study aimed to determine whether imaging assessments can detect microvascular effects of a mixed meal tolerance test (MMTT).

**Methods:**

Twenty healthy volunteers (age ≥45 and ≤70 years) underwent two MMTTs at the beginning (Day 1) and end (Day 84) of a twelve‐week period. Imaging methods included laser speckle contrast imaging (LSCI) combined with post‐occlusive reactive hyperaemia (PORH) and local thermal hyperaemia (LTH) challenges, passive leg movement ultrasonography (PLM), and sidestream dark field microscopy (SDFM). Measurements were conducted pre‐MMTT and at 5 timepoints post‐MMTT for PLM and SDFM and 3 timepoints post‐MMTT for PORH and LTH.

**Results:**

No consistent effects of the MMTT were detected on LSCI LTH, PLM and SDFM endpoints. LSCI PORH maximum perfusion was significantly suppressed 46, 136, and 300 min post‐MMTT administration on Day 1, while residual perfusion decreased significantly 46 and 136 min post‐MMTT on Day 1. However, when repeated on Day 84, PORH endpoints were not significantly affected by the MMTT.

**Conclusion:**

SDFM, PLM and LSCI LTH endpoints displayed high intra‐subject variability and did not detect consistent effects of MMTT. LSCI PORH endpoints displayed the lowest intra‐subject variability of all assessed endpoints and were affected by the MMTT on Day 1, but not on Day 84. Further standardization of methods or more robust challenges to affect vascular endpoints may be needed.

## 1 INTRODUCTION

The vascular endothelium plays a central role in maintaining homeostasis in the cardiovascular system. Endothelial cells regulate vascular tone, promote restoration of damaged vessels, inhibit excessive coagulation, and aid inflammatory and immunological responses (Daiber et al., 2017). Dysfunction of the endothelium is characterized by a reduction in nitric oxide (NO) bioavailability. The lack of vasodilatory effects of NO and thereby a relative overabundance of endothelial constrictive factors, hampers the function of the endothelium. This dysfunctional state is regarded as a risk factor for and cause of cardiovascular disease (Gimbrone & Garcia‐Cardena, 2016). Endothelial dysfunction is correlated to traditional cardiovascular risk factors such as smoking, dyslipidaemia and hypertension and associated with worse outcomes in cardiovascular disease (Bonetti et al., 2003; Constans & Conri, 2006; Higashi et al., 2009). Endothelial dysfunction is also associated with metabolic disturbances in the form of insulin resistance and diabetes mellitus type 2, (Polovina & Potpara, 2014) and evidence mounts that endothelial cells are actively involved in metabolic homeostasis (Pi et al., 2018). Proposed mechanisms for endothelial dysfunction in metabolic disturbance include increased oxidative stress and inflammation, increased exposure to advanced glycation end‐products, and lipotoxicity (Pi et al., 2018; Polovina & Potpara, 2014).

In healthy humans, endothelial function is assumed to be optimal, and hence improvement by intervention unlikely, complicating the detection of treatment effects on endothelial function. However, this normal physiological balance can be disturbed by a metabolic challenge, such as a mixed meal tolerance test (MMTT), also termed “PhenFlex” test (van den Broek et al., 2017; Wopereis et al., 2017). This MMTT can induce metabolic disturbance in the form of hyperlipidemia (Neumann & Egert, 2022) and hyperglycemia (Al Balwi et al., 2022), affecting endothelial function through several pathways (Kim et al., 2012; Meza et al., 2019; Williams et al., 1998). Although evidence of induction of endothelial dysfunction by a glucose load is mixed, with evidence for deterioration of endothelial function after an oral glucose load (Major‐Pedersen et al., 2008; Weiss et al., 2008), but improvement after intravenously induced hyperglycemia (Horton et al., 2022), meals with high fat content consistently attenuate endothelial function in healthy volunteers (Fewkes et al., 2022; Steer et al., 2003). The response to MMTT is usually only evaluated using blood‐based biomarkers or the flow‐mediated dilation (FMD) technique (Hoevenaars et al., 2019), which measures the NO‐dependent vasodilatory response of the endothelium to shear stress, and is known to decrease after ingestion of meals (Thom et al., 2016).

The addition of other imaging assessments besides FMD may provide additional information on pathways involved in endothelial (dys)function, such as axon reflexes, cyclo‐oxygenase function, and vascular angiogenesis, as well as different vascular beds. The additional methods investigated in this study include laser‐speckle contrast imaging (LSCI) combined with local thermal hyperaemia (LTH) and post‐occlusive reactive hyperaemia (PORH) challenges, passive leg movement (PLM) ultrasonography and sidestream dark field microscopy (SDFM). These techniques capture various physiological pathways involved in endothelial function. Different phases of the response to LTH capture both NO‐dependent vasodilation (Brunt & Minson, 2012; Cracowski & Roustit, 2016), as well as the role of the role of axon reflexes (Minson, 2010). The increase in femoral artery flow during PLM as measured with doppler ultrasonography is 80‐90% NO‐dependent (Groot et al., 2015; Mortensen et al., 2012; Trinity et al., 2012), while changes in PORH response follow different physiological pathways, with involvement of cyclo‐oxygenase derived prostanoids, sensory nerves, and endothelial‐derived hyperpolarizing factors (Hellmann et al., 2015). The imaging techniques also assess different vessel sizes, as LSCI measures skin microcirculation up to approximately 700 µm depth, (Davis et al., 2014) PLM measures large (femoral) artery blood flow, and SDFM measures density and blood flow in the sublingual microcirculation, thereby deriving a measure of vessel growth and patency. The combination of these different techniques can therefore provide mechanistic insights in endothelial function on several levels. In combination with a challenge that can induce a change in endothelial function, the spectrum of endothelial physiology and effects of intervention on it can then be measured.

In this validation study, the variability and ability to detects of an MMTT of the aforementioned imaging methods were investigated to examine their usefulness in the assessment of endothelial function. The investigated imaging techniques could then potentially be used in the assessment of investigational medical products aimed at reducing or preventing endothelial dysfunction, e.g., cardiovascular risk management drugs.

## 2 METHODS

This study was conducted at Centre for Human Drug Research in accordance with the principles of the Declaration of Helsinki, the International Council on Harmonization Good Clinical Practice, and ethical principles as referenced in EU Directive 2001/20/EC and was approved by an independent ethics committee (BEBO, Assen) before study execution. The study was performed as part of a larger study investigating the effects of specific dietary fibres on gut microbiome and resilience. The results of this investigation are out of scope for the aims of this paper and described elsewhere (Eveleens Maarse et al., 2024; van den Broek et al., 2017). All participants signed an Informed Consent Form before any study‐related procedures were conducted. The trial was registered in the Dutch clinical trial registry toetsingonline. nl (NL71723.056.19) and at clinicaltrials. gov (NCT04829396).

### 2.1 Study design

Twenty participants were planned to be included in the study, which aimed to characterize the physiological response of the endothelium to an MMTT. Participants underwent an MMTT during an ambulatory visit at the start of the study to identify treatment‐naïve responses to MMTT in the assessed endpoints. Then, as part of the overarching study, participants received the study treatment or matching placebo once daily for 12 weeks, randomized in a 1:1 ratio. At the end of the 12‐week treatment period, an MMTT was administered again during an ambulatory visit to the study center, i.e., on Day 84. Only Day 84 data from participants treated with placebo are included in the analyses of this sub‐study to avoid confounding treatment effects and characterize only the innate endothelial response to meals.

Pre‐MMTT on Day 1 and Day 84, all endothelial measurements were conducted to establish a baseline. Post‐MMTT, endothelial measurements were repeated following the schedule depicted in Table 1 and Figure S1. A complete list of endpoints analysed per imaging technique is provided in Supplemental Table S1.

**Table 1:** Schedule of imaging assessments and pharmacodynamic blood sampling before and after mixed meal tolerance test (zero point) on Day 1 and Day 84.

	−30 min	30 min	60 min	120 min	240 min	300 min	330 min
**LSCI LTH**	X		X		X		X
**LSCI PORH**	X	X		X		X	
**PLM**	X	X	X	X	X		X
**SDFM**	X	X	X	X	X		X

Abbreviations: LSCI, laser speckle contrast imaging; LTH, local thermal hyperemia; PLM, passive leg movement; PORH, post‐occlusive reactive hyperemia; SDFM, sidestream dark field microscopy.

### 2.2 Study population

Adult (age ≥45 and ≤70 years) male and female participants were eligible for inclusion if no clinically significant abnormal findings were obtained during a medical screening, which included medical history, physical examination, 12‐lead ECG, alcohol breathalyzer, and clinical laboratory tests (i.e., serum chemistry, hematology, coagulation, urine drug screen, and urinalysis). Participants using any type of medication or dietary supplements in the 7 days before study start, with the exception of paracetamol up to 4 g/day and ibuprofen up to 1 g/day, or history of use of antibiotics, antacids, laxatives, statins, antidiarrheal, immunomodulatory or antidiabetic medication <3 months before the start of study were excluded, as well as participants with documented food allergies, a history of multiple severe drug allergies and participants with a vegan, macrobiotic or a medically prescribed diet.

### 2.3 Sample size justification

The study aim was exploratory in nature and therefore no formal power calculation was performed, although sample sizes of 10 to 20 subjects are generally accepted in early phase clinical trials employing explorative pharmacodynamic endpoints (Rubinstein et al., 2010).

### 2.4 Mixed‐meal tolerance test (MMTT)

During the ambulatory visits, the MMTT was performed in the morning after participants fasted for at least 10 h. The challenge consisted of 500 mL mixed‐meal challenge drink, containing a mixture of 12.40% (w/w) palm olein, 17.25% (w/w) dextrose, 4.13% (w/w) Protifar (Nutricia), 0.10% (w/w) (vanilla flavor), 0.12% (w/w) trisodiumcitrate, 0.08% (w/w) sodiumhydroxide, and 66.12% (w/w) water, resulting in a caloric intake of 950 kcal with 60 g fat (of which 39% saturated fatty acids, 47% mono unsaturated fatty acids, 14% poly unsaturated fatty acids), 75 g glucose, and 20 g protein. The challenge drink was composed as described in literature (produced by ‘Instituut voor Landbouw‐ Visserij‐ en Voedingsonderzoek, Eenheid Technologie & Voeding, Food Pilot’, Melle, Belgium) (Wopereis et al., 2017). MMTT challenge drinks were stored in a refrigerator with restricted access at 2–8°C until dispensing and were to be completely consumed by participants within 5 min of dispensing (Hoevenaars et al., 2019).

### 2.5 Study assessments

#### 2.5.1 Biochemical parameters

Plasma levels of aspartate‐amino‐transferase, alanine‐amino‐transferase, glucose, high‐density lipoprotein (HDL) cholesterol, low‐density lipoprotein (LDL) cholesterol, ratio HDL/LDL cholesterol, insulin, C‐reactive protein, and gamma‐glutamyltransferase and cytokines (interleukin [IL]‐1β, IL‐2, IL‐4, IL‐6, IL‐8, IL‐10, IL‐12p70, IL‐13, interferon‐γ, tumor necrosis factor) were measured at baseline and during the MMTT test. Results of these analyses are described elsewhere (Eveleens Maarse et al., 2024).

#### 2.5.2 Laser speckle contrast imaging (LSCI)

LSCI is a technique used to determine superficial blood flux in the skin by comparing changes in speckle contrast, creating a measure of movement of red blood cells expressed in arbitrary units (Bezemer et al., 2010). Cutaneous microcirculation was assessed through LSCI using a dedicated laser speckle imager (PeriScan PSI system, Perimed, Jäfälla, Sweden). The characteristics of LSCI measurements have been discussed in the literature (Cracowski & Roustit, 2010; Heeman et al., 2019; Roustit & Cracowski, 2012) and were described in the clinical study protocol. All LSCI imaging was conducted on the same arm for each subject throughout the study.

#### 2.5.3 Local thermal heating

After fixation of the arm in a sand cushion, a round heating probe 1 cm in diameter (moorVMS‐HEAT, Moor Instruments, Axminster, United Kingdom) was placed on the forearm and filled with lukewarm water. Basal cutaneous perfusion was measured through this probe from a distance of approximately 15 cm for 5 min, after which the probe was heated to 43°C and skin perfusion response to heating as measured by LSCI was assessed for 20 min. After the measurement, the region of interest was defined as inside the inner circumference of the heating probe, and 3 time periods of interest were defined: start of measurement—300 s = basal perfusion period, 420 s – 500 s = maximum perfusion period and 780 s – end of measurement = plateau perfusion period.

#### 2.5.4 Post‐occlusive reactive hyperaemia (PORH)

A blood pressure cuff was placed around the upper arm, after which forearm cutaneous perfusion was measured in an area of 10 by 4 cm from a distance of approximately 15 cm. After 5 min of measurement, the blood pressure cuff was inflated to >50 mmHg above systolic blood pressure for 5 min and subsequently released, recording respectively 5‐min basal, 5‐min occlusion and 5‐min post‐occlusion cutaneous perfusion data as measured with LSCI. After the measurement, the region of interest was defined as the entire measured area and 4 time periods of interest were defined: start of measurement – 300 s = basal perfusion period, 300 s–600 s = occlusion perfusion period, 600 s–630 s = maximum perfusion period, and 630 s – end of measurement = post‐occlusive residual perfusion period.

#### 2.5.5 Single passive leg movement (PLM)

Single PLM is a technique for assessing vasodilation in the lower leg in response to movement of the lower leg. The diameter of the common femoral artery was measured using ultrasonography while the leg was stretched in a horizontal position. With continuous doppler flowmetry, videos were obtained of the velocity of blood flow through the femoral artery for 30 s as a baseline measurement. Then, the leg was bent and stretched 90° to induce lower limb vasodilation. The doppler flowmetry continued for 180 s after movement. Custom automatic video analysis software (Van Stein & Groentjes, Leiden, The Netherlands) extracted time averaged maximum velocity of blood flow for every heart cycle in the measurement. Blood flow volume in mL/min was then calculated with the formula $\mathrm{Flow}=\pi r^{2}\;\times v\times 60$ in which *r* is the femoral artery radius in cm and *v* flow velocity in cm/s. Time periods of interest were defined as start of measurement – 30 s = baseline flow, 30 s–60 s = peak blood flow and 60 s – end of measurement = residual blood flow.

#### 2.5.6 Sidestream dark field microscopy (SDFM)

The SDFM technique uses light in a wavelength absorbed by red blood cells penetrating 0.5 mm into the imaged tissues, allowing for visualization of red blood cells and vessels in the surface layer of that tissue as black opacities (D. H. Lee et al., 2014; Rovas et al., 2019). Sublingual microvascular function was assessed by SDFM imaging with a handheld microcirculation scanner (MicroScan, MicroVision Medical, Amsterdam, The Netherlands). At each timepoint, five 300‐frame videos with a framerate of 60 Hz were obtained of the sublingual vasculature and the following parameters were calculated using AVA5 software (MicroVision Medical, Amsterdam, The Netherlands): number of vessel crossings, DeBacker density, number of small vessel crossings, small vessel DeBacker density, perfused number of vessel crossings, perfused DeBacker density, perfused number of small vessel crossings, perfused small vessel DeBacker density, consensus proportion perfused vessels (PPV) and consensus PPV for small vessels.

### 2.6 Statistical analysis

All statistical analyses were performed according to a statistical analysis plan written before database lock. All safety and statistical programming was conducted with SAS 9.4 for Windows (SAS Institute Inc., Cary, NC, USA).

Continuous demographic variables (e.g., age, height, weight, BMI) were summarized by descriptive statistics (n, mean, SD, median, Min, Max). Qualitative demographic characteristics (sex, race/ethnicity) were summarized by counts and percentages.

The analysis population for MMTT response tests on Day 1 was defined as all participants who received the MMTT and had at least one post‐baseline assessment of the analyzed parameter. On Day 84, only subjects treated with placebo were included in the analysis population. All repeatedly measured endothelial parameters were summarized (n, mean, SD, median, minimum, and maximum values) by treatment and time, with treatment “none” representing measurements before the start of intervention, i.e., all Day 1 measurements. Least squares means (LSMs) of the change from baseline (CFB), with baseline defined as the first pre‐MMTT imaging assessment, and 95% confidence intervals (CI) were calculated using a linear mixed model for the ‘placebo’ and ‘none’ treatment groups for each timepoint. Intrasubject variability was assessed by calculating the coefficient of variation (CV%) between Day 1 and Day 84 measurements for placebo subjects who received both MMTTs using the within‐subject standard deviation method (Synek, 2008), i.e., calculating the individual standard deviation divided by the mean. An ICC(1,1) model was used to determine the test/retest reliability by calculating intraclass correlation coefficients and their 95% confidence intervals. (Koo and Mae, 2016).

## 3 RESULTS

### 3.1 Participant disposition

A total of 21 participants were enrolled in the endothelial assessment sub‐study, one more than originally planned due to drop‐out of one participant after the first MMTT on Day 1 due to antibiotic use. Hence, 21 participants were included in the analysis of treatment‐naïve effects of MMTT on Day 1. Ten participants were treated with placebo for 12 weeks and included in the analysis of intra‐subject variability.

Baseline characteristics of the study population are shown in Table 2. No clear differences between participants assigned to placebo versus all participants were identified, hence group characteristics on Day 1 and Day 84 were comparable. Reproducibility.

**Table 2:** Baseline characteristics of study participants on Day 1 and 84 before MMTT administration.

Parameter [Unit]	Day 1 (All) (n = 21)	Day 84 (n = 10)
Sex [% male]	67	73
Age [Years] mean ± SD	59.4 ± 5.9	62.9 ± 4.6
Body weight [kg] mean ± SD	87.4 ± 11.1	85.6 ± 8.8
Body mass index [kg/m^2] mean ± SD	27.7 ± 1.5	27.4 ± 1.9
Systolic BP [mmHg] mean ± SD	129.5 ± 17.1	133.1 ± 21.2
Diastolic BP [mmHg] mean ± SD	74.8 ± 8.1	76.9 ± 9.5
Heart rate [bpm] mean ± SD	67.0 ± 9.6	66.3 ± 9.8
Hip circumference [cm] mean ± SD	105.3 ± 7.8	104.7 ± 10.3
Waist circumference [cm] mean ± SD	97.3 ± 9.4	98.3 ± 9.9

Abbreviations: BP, blood pressure; MMTT, mixed meal tolerance test; SD, standard deviation.

A summary of reproducibility (ICC and CV%) assessments is shown in Table 3.

**Table 3:** Summary of intraclass correlation coefficients with 95% CI and CV% per imaging endpoint.

Endpoint	ICC(1,1)	95% CI Lower	95% CI Upper	CV (%)
LTH Maximum perfusion	0.08	−0.16	0.30	26.6
LTH Plateau perfusion	0.32	0.10	0.52	23.6
PORH Maximum perfusion	0.56	0.37	0.70	6.9
PORH Residual perfusion	0.43	0.22	0.60	9.1
PLM Peak flow	0.21	‐0.09	0.47	18.1
PLM Total flow	0.39	0.16	0.59	17.0
PLM CFB flow	0.31	0.10	0.49	40.1
SDFM DeBacker density	0.15	0.04	0.26	20.1
SDFM Perfused DeBacker density	0.14	0.02	0.25	21.5
SDFM Number of crossings	0.04	−0.08	0.15	14.0
SDFM Consensus PPV	0.03	−0.14	0.08	5.6

Abbreviations: CFB, change from baseline; CI, confidence interval; CV%, coefficient of variation; LSCI, laser speckle contrast imaging; LTH, local thermal hyperemia PLM, passive leg movement; PORH, post‐occlusive reactive hyperemia; SDFM, sidestream dark field microscopy.

### 3.2 Laser speckle contrast imaging

#### 3.2.1 Local thermal hyperaemia

**Figure 1:**
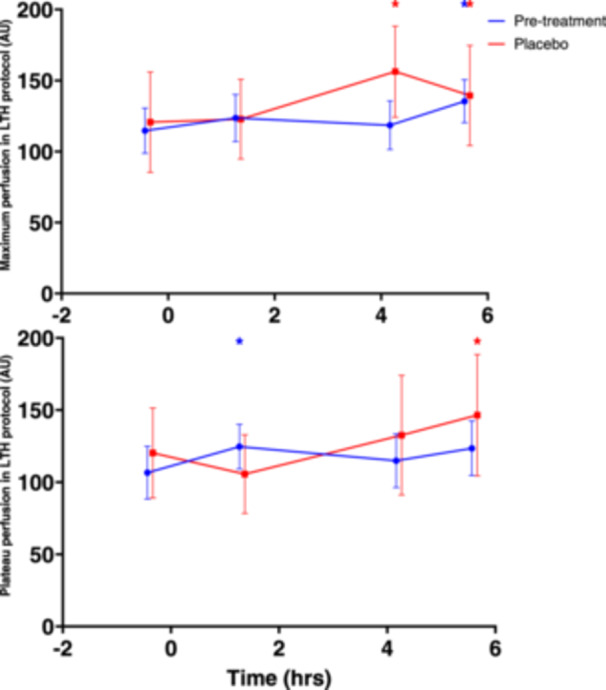
Effect of MMTT on LSCI LTH parameters. Mean (SD) of maximum (upper panel) and plateau (lower panel) perfusion in LSCI LTH protocol before and after MMTT on Day 1 (treatment = none) and Day 84 (placebo only). MMTT was administered at time (hrs) = 0. Timepoints with statistically significant difference from baseline marked with *. Abbreviations: AU = arbitrary units; LTH = local thermal hyperaemia; LSCI = laser speckle contrast imaging; MMTT = mixed meal tolerance test; SD = standard deviation.

Maximum and plateau perfusion during LSCI LTH protocol before and after performance of MMTT are shown in Figure 1. No consistent statistically significant changes after MMTT administration occurred (Table 4).

**Table 4:** LSMs CFB with 95% CI of maximum perfusion and plateau perfusion during LTH protocol on study Day 1 and 84. Statistically significant changes (*p* < 0.05) from pre‐MMTT values bolded.

			95% CI	
Treatment	Time post‐MMTT (h:mm)	LSM CFB	Lower	Upper
Maximum perfusion				
None (Day 1)	1:22	9.564	−5.755	24.884
	4:16	4.375	−10.944	19.695
	5:40	**21.125**	**5.805**	**36.444**
Placebo (Day 84)	1:22	9.698	−14.464	33.860
	4:16	**40.581**	**17.290**	**63.871**
	5:40	**23.998**	**0.707**	**47.288**
Plateau perfusion				
None (Day 1)	1:22	**16.460**	**0.821**	**32.099**
	4:16	6.907	−8.732	22.546
	5:40	15.264	−0.375	30.903
Placebo (Day 84)	1:22	−4.214	−29.055	20.628
	4:16	17.087	−6.635	40.809
	5:40	**33.707**	**9.984**	**57.429**

Abbreviations: CFB, change from baseline; CI, confidence interval; LSM, least squares means; LTH, local thermal hyperaemia; MMTT, mixed meal tolerance test.

#### 3.2.2 Post occlusive reactive hyperaemia

**Figure 2:**
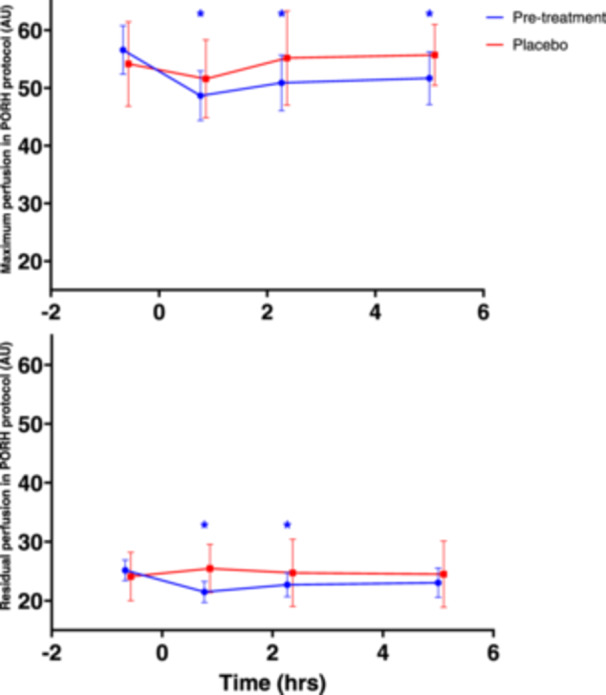
Effect of MMTT on LSCI PORH parameters. Mean (SD) of maximum (upper panel) and residual (lower panel) perfusion in LSCI PORH protocol before and after MMTT on Day 1 (treatment = none) and Day 84 (placebo only). MMTT was administered at time (hrs) = 0. Timepoints with statistically significant difference from baseline marked with *. Abbreviations: AU = arbitrary units; LSCI = laser speckle contrast imaging; MMTT = mixed meal tolerance test; PORH = post occlusive reactive hyperemia; SD = standard deviation.

Results of the PORH endpoints maximum perfusion and residual perfusion before and after MMTT administration on Day 1 and Day 84 are shown in Figure 2 and Table 5.

**Table 5:** LSMs CFB with 95% CI of maximum perfusion and residual perfusion during PORH protocol on study Day 1 and 84. Statistically significant changes (*p* < 0.05) from pre‐MMTT values bolded.

			95% CI	
Treatment	Time post‐MMTT (h:mm)	LSM CFB	Lower	Upper
Maximum perfusion				
None (Day 1)	0:46	**−8.328**	**−11.378**	**−5.278**
	2:16	**−5.983**	−**9.033**	**−2.934**
	5:00	**−5.052**	−**8.102**	**−2.002**
Placebo (Day 84)	0:46	**−**3.888	**−**8.751	0.976
	2:16	**−**0.538	**−**5.208	4.132
	5:00	**−**0.587	**−**5.257	4.084
Residual perfusion				
None (Day 1)	0:46	**−3.775**	−**6.028**	**−1.523**
	2:16	**−2.546**	**−4.798**	**−0.293**
	5:00	**−**2.185	**−**4.438	0.068
Placebo (Day 84)	0:46	**−**1.265	**−**4.560	2.030
	2:16	**−**0.871	**−**4.097	2.355
	5:00	**−**1.039	**−**4.265	2.187

Abbreviations: CFB, change from baseline; CI, confidence interval; LSM, least squares means; MMTT, mixed meal tolerance test; PORH, post‐occlusive reactive hyperaemia.

Maximum perfusion after PORH decreased significantly 46, 136 and 300 min post‐MMTT administration in all participants on Day 1 (LSMs CFB −8.328, 95% confidence interval (CI): −11.378, −5.278, −5.983, 95% CI: −9.033, −2.934 and −5.052, 95% CI: −8.102, −2.002, respectively). On Day 84, maximum perfusion after PORH did not decrease significantly. Residual perfusion after PORH also decreased significantly 46 and 136 min post‐MMTT in all participants on Day 1 (LSMs CFBs −3.775, 95% CI: −6.028, −1.523 and −2.546, 95% CI: −4.798, −0.293, respectively). On Day 84, this decrease was not significant, similar to the findings for maximum perfusion.

### 3.3 Single passive leg movement

**Figure 3:**
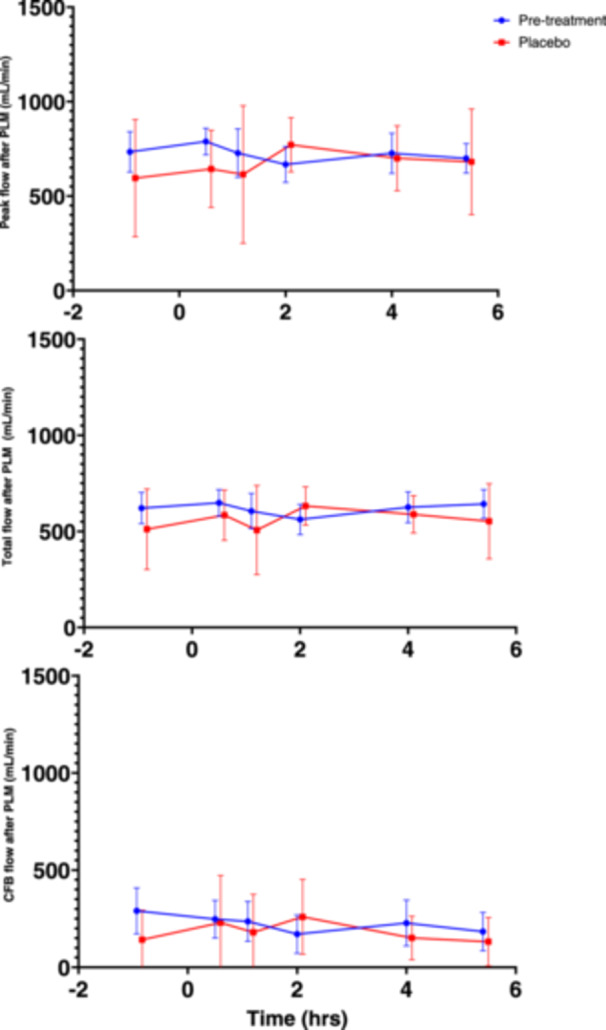
Effect of MMTT on PLM parameters. Mean (SD) of peak (upper panel), total (middle panel) and CFB (lower panel) flow in the femoral artery after PLM, before and after MMTT on Day 1 and Day 84 (placebo only). MMTT was administered at time (hrs) = 0. Timepoints with statistically significant difference from baseline marked with *. Abbreviations: CFB, change from baseline; MMTT, mixed meal tolerance test; PLM, passive leg movement; SD, standard deviation.

The results of PLM testing pre‐ and post‐MMTT are shown in Figure 3. No consistent statistically significant changes after MMTT administration occurred on Day 1 (Table S2).

### 3.4 Sidestream dark field microscopy

**Figure 4:**
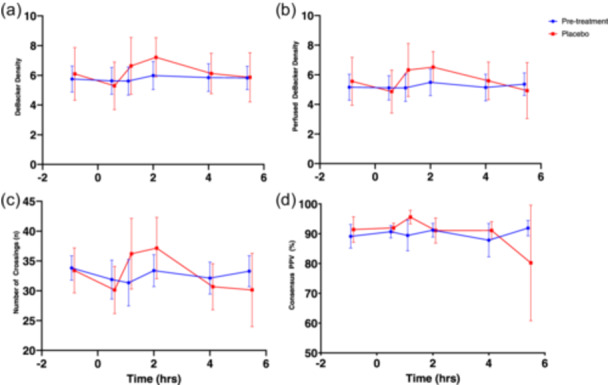
Effect of MMTT on SDFM parameters. Mean (SD) of DeBacker density (panel A), perfused DeBacker density (panel B), number of crossings (panel C) and Consensus PPV (panel D) before and after MMTT on Day 1 and 84 (placebo only). MMTT was administered at time (hrs) = 0. Timepoints with statistically significant difference from baseline marked with *. Abbreviations: MMTT, mixed meal tolerance test; PPV, proportion perfused vessels; SD, standard deviation.

SDFM parameters before and after administration of MMTT on Day 1 and 84 are shown in Figure 4. No consistent statistically significant changes after MMTT administration occurred on Day 1.

## 4 DISCUSSION

This study assessed the variability of several imaging techniques and whether these techniques could detect changes in endothelial function caused by a MMTT. In addition, the variability of MMTT‐induced changes over time was evaluated. Overall, LSCI combined with PORH had the lowest intrasubject CV% with 6.9% and 9.1% for peak and residual perfusion, respectively, which is slightly lower than the CV% for FMD measured in previous studies (9.9 ± 8.4%) (Ghiadoni et al., 2012). This indicates that the LSCI PORH protocol may have comparable reproducibility, although the comparison favors LSCI since this assessment is measured in absolute values as opposed to percent change in FMD. Maximum perfusion measured in the LSCI PORH protocol showed an ICC of 0.56, corresponding with ‘moderate’ reliability, supporting the comparison to FMD (Koo & Mae, 2016). LSCI combined with LTH, PLM and most SDFM parameters showed high intrasubject variability with CV% ranging from 14.0% to 40.1% and ICC < 0.5. SDFM PPV showed a low CV% but also low ICC, possibly since the percentage of perfused vessels in healthy volunteers is almost always close to 100% but varies within the individual.

LSCI PORH was the only assessed imaging modality that detected significant decreases in endpoints after MMTT administration on Day 1, likely due to its low intra‐subject variability. This may have been caused by a reduction of endothelial function through induction of low‐grade inflammation and oxidative stress (Aljada et al., 2004; Emerson, Sciarrillo, Kurti, Emerson, & Rosenkranz, 2018; I. K. Lee et al., 2002; Weiss et al., 2008). PORH is a relatively broad measure of vascular function, as it can be influenced by a range of factors including a high‐salt diet (Cavka et al., 2015), cyclo‐oxygenase inhibition (Medow et al., 2007), nitric oxide inhibition (Dakak et al., 1998) and neuronal blockade (McGarr & Cheung, 2022), although evidence is mixed (Hellmann et al., 2015; Wong et al., 2003). PORH therefore may reflect overall (micro)vascular function and ability of the endothelium to respond to stimuli, in particular shear stress (Balasubramanian et al., 2021), without pointing to specific pathways involved in vasodilation. Maximum and residual perfusion during and after PORH were significantly reduced after administration of a mixed meal on Day 1, but not on Day 84, potentially due to the smaller sample size (*n* = 10) on study Day 84 and the limited magnitude of the detected effects. This is supported by the fact that response curves on Day 1 and Day 84 show similar patterns.

The effects of meals on vascular function as assessed with FMD have been well characterized. FMD decreases after ingestion of both mixed meals and a glucose challenge, and this effect is modulated by BMI, sex and importantly cardiometabolic disease status. Assessment of post‐prandial FMD has therefore been suggested as a method of identifying individuals at risk for cardiometabolic disease (Thom et al., 2016). FMD and LSCI PORH measure similar physiological processes since both employ an occlusion‐reperfusion challenge. However, the LSCI PORH technique does not suffer from the drawbacks associated with FMD, such as technical complexity and high operator‐dependence (Thijssen et al., 2011). In addition, while FMD measures larger arteries, LSCI PORH is limited to assessment of superficial skin microcirculation. This vascular bed can be differently affected by various challenges, such as the MMTT. The imaging techniques may therefore complement each other. It is however advisable to include larger sample sizes than employed in this study for the assessment of effects of LSCI PORH, since the effect of the MMTT on occlusion‐reperfusion did not rise to statistical significance on Day 84.

The other measures of vascular function, i.e., LSCI combined with LTH, PLM, and SDFM showed high variability and no clear response to the mixed meal test in this study. The intrasubject CV% for PLM and LSCI LTH in this study were comparable to those found in earlier studies, while those for SDFM were substantially higher (Groot et al., 2022; Gu et al., 2015; Roustit et al., 2010). The high intrasubject CV% combined with the small sample size of this study may have increased the chance for type II error for these techniques, limiting the ability of these imaging methods to detect effects of the MMTT. Moreover, for SDFM, the time period of the assessments performed post‐MMTT may have been too short to allow assessment of long‐term effects of a glucose and fat load on angiogenesis. Alternatively, vascular function as measured with PLM, SDFM or LSCI LTH might not be affected to a large enough extent by the MMTT to be detected in a study with the current sample size. Although the MMTT may not have been robust enough to modulate NO‐dependent vasodilation as measured with PLM and LTH or angiogenesis as measured with SDFM acutely (Bakker et al., 2009; Okon et al., 2007), meal effects were detected by the more sensitive LSCI PORH technique, but not consistently. Therefore, employing a challenge known to significantly and acutely affect microcirculation and endothelial function, such as lipopolysaccharide infusion (Dillingh et al., 2014), may be advisable when examining effects of pharmacological interventions using the less sensitive SDFM, PLM or LSCI LTH modalities. Combining these techniques with hyper‐ or euglycemic and hyper‐ or euinsulinemic clamps (Perkins et al., 2015) to isolate specific physiological mechanisms (Scherrer et al., 1994) may also create opportunities to detect metabolic effects on the vasculature more precisely.

## 5 LIMITATIONS

This study was part of a larger study aiming to assess the effects of a 12‐week dietary intervention on MMTT response and gut microbiome. Hence, the study design was not optimized for the objectives described in this sub‐study. Specifically, no control group undergoing assessments without MMTT administration was included, limiting the statistical possibilities to determine effects of the challenge on the assessed parameters, as changes post MMTT administration could be due to diurnal or random variation. Additionally, due to the MMTT challenge, repeated measures on the same day as an assessment of method consistency were not possible in this study. Since the effects of the MMTT on occlusion‐reperfusion were not consistent across the study period, the likelihood that factors other than MMTT mediated the effects measured on Day 1 increases. Alternatively, the lack of significant effects on occlusion‐reperfusion on Day 84 can be explained by the reduction of sample size and hence statistical power, since participants receiving active treatment in the overarching study were excluded from Day 84 analysis. Another limitation of the present study is that the calculation of intrasubject variability was based on repeated measurements in the placebo group over a 12‐week period of time and may therefore have been influenced by the placebo intervention. Finally, this study did not include FMD or blood‐based biomarkers in its design, limiting the possibilities to benchmark the measured effects against findings in the literature. In future studies, the employed imaging techniques should be validated by comparison with FMD.

## 6 CONCLUSION

Significant effects of MMTT administration on occlusion‐reperfusion response were identified in this study. However, these effects were not consistent over the 3‐month study period, possibly due to a smaller sample size at the 3‐month timepoint. Occlusion‐reperfusion as assessed with LSCI showed reproducibility comparable with FMD. All other assessed imaging modalities showed high variability, poor reliability and did not detect effects of the MMTT. Hence, based on the results obtained in this study, most imaging assessments are not able to detect microvascular effects of an MMTT, and further research is needed to investigate the extent to which LSCI may be able to detect these microvascular effects and possibly refine the measurement protocol to further improve reliability.

## CONFLICTS OF INTEREST STATEMENT

IW is an employee of dsm‐firmenich and may hold shares and/or stock options in this company. All other authors declare no competing interests for this work.

## Supporting information

Supporting information.

Supporting information.

Supporting information.

Supporting information.

## Data Availability

The data generated in this trial are not publicly available due to legal restrictions. However, the corresponding author can be approached for more detailed insights into the presented graphs and tables.
