# Supplementary material for: Microvascular effects of a mixed meal tolerance test: a model validation study
Source: Clin Physiol Funct Imaging. 2024 Sep 23;45(1):e12904. doi: 10.1111/cpf.12904 (PMC11650408; doi:10.1111/cpf.12904)
Supplement: Supplementary file 1 — Supporting information. [file CPF-45-0-s003.pdf]

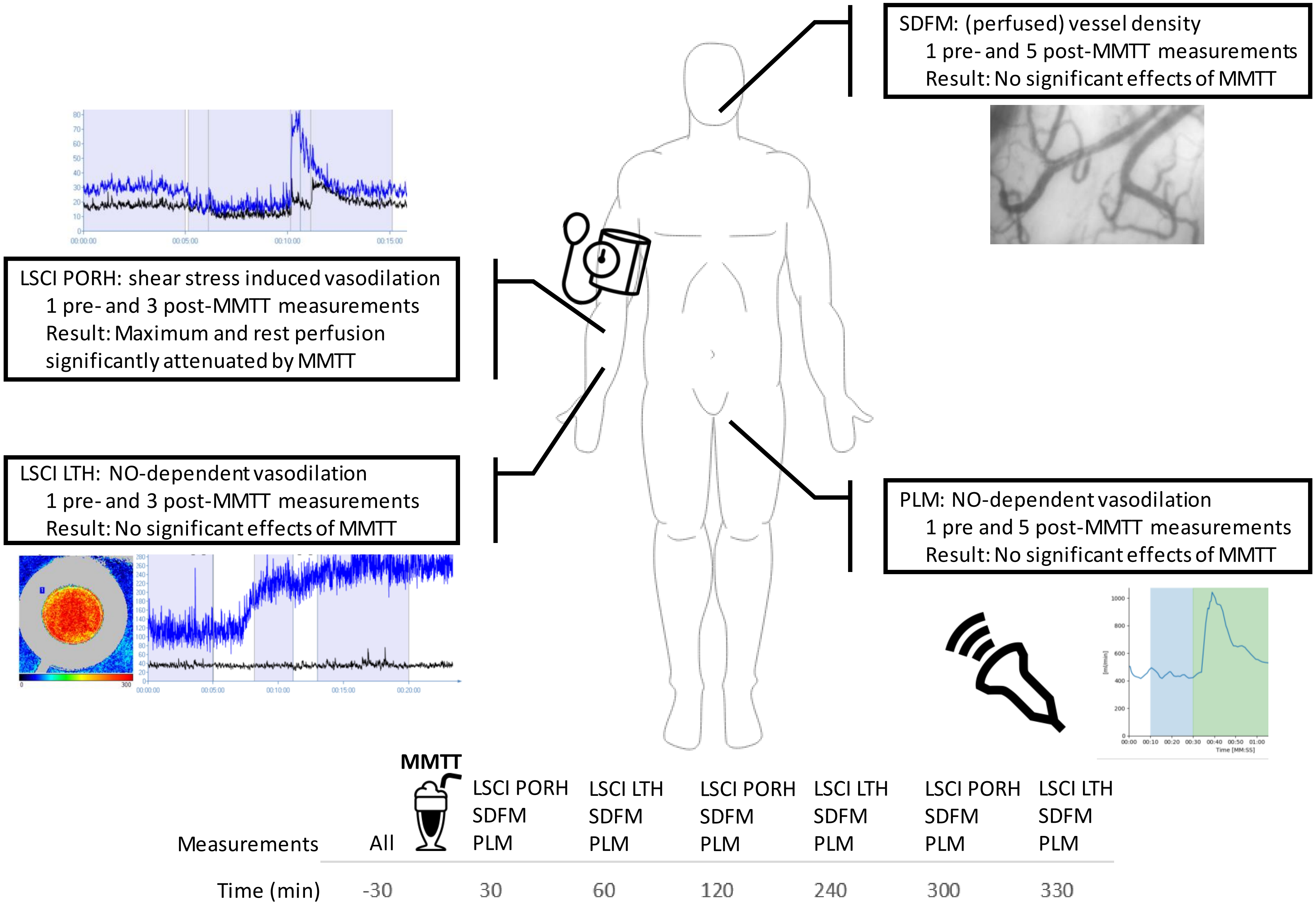
SDFM: (perfused) vessel density
1 pre- and 5 post-MMTT measurements
Result: No significant effects of MMTT
LSCI PORH: shear stress induced vasodilation
1 pre- and 3 post-MMTT measurements
Result: Maximum and rest perfusion significantly attenuated by MMTT
LSCI LTH: NO-dependent vasodilation
1 pre- and 3 post-MMTT measurements
Result: No significant effects of MMTT
PLM: NO-dependent vasodilation
1 pre and 5 post-MMTT measurements
Result: No significant effects of MMTT
MMTT
Measurements
All
LSCI PORH SDFM PLM
LSCI LTH SDFM PLM
LSCI PORH SDFM PLM
LSCI LTH SDFM PLM
LSCI PORH SDFM PLM
LSCI LTH SDFM PLM
Time (min)
-30
30
60
120
240
300
330
