# Supplementary material for: Microvascular effects of a mixed meal tolerance test: a model validation study
Source: Clin Physiol Funct Imaging. 2024 Sep 23;45(1):e12904. doi: 10.1111/cpf.12904 (PMC11650408; doi:10.1111/cpf.12904)
Supplement: Supplementary file 2 — Supporting information. [file CPF-45-0-s002.docx]

| Assessment | Endpoints |
| --- | --- |
| LSCI LTH | Maximum perfusion, plateau perfusion |
| LSCI PORH | Maximum perfusion, rest perfusion |
| PLM | Peak blood flow, total blood flow, CFB flow |
| SDFM | Perfused DeBacker density, perfused small vessel DeBacker density, consensus PPV, consensus PPV small |

Table S1: Endpoints analysed per imaging assessment. Abbreviations: CFB = change from baseline; LSCI = laser speckle contrast imaging; LTH = local thermal hyperemia PLM = passive leg movement; PORH = post-occlusive reactive hyperemia; PPV = proportion perfused vessels; SDFM = side-stream dark field microscopy.
