# Supplementary material for: Microvascular effects of a mixed meal tolerance test: a model validation study
Source: Clin Physiol Funct Imaging. 2024 Sep 23;45(1):e12904. doi: 10.1111/cpf.12904 (PMC11650408; doi:10.1111/cpf.12904)
Supplement: Supplementary file 3 — Supporting information. [file CPF-45-0-s004.docx]

|  | | | 95%CI | |
| --- | --- | --- | --- | --- |
| **Treatment** | **Time post-MMTT** | **LSM CFB** | **Lower** | **Upper** |
| **Total flow** | | | | |
| None (Day 1) | 0:36 | 36.266 | -44.908 | 117.440 |
|  | 1:12 | 16.387 | -61.296 | 94.070 |
|  | 2:06 | -29.244 | -110.740 | 52.253 |
|  | 4:06 | 71.469 | -9.805 | 152.742 |
|  | 5:30 | 51.619 | -28.077 | 131.314 |
| Placebo (Day 84) | 0:36 | -6.523 | -171.639 | 158.593 |
|  | 1:12 | 2.319 | -144.628 | 149.266 |
|  | 2:06 | 98.408 | -48.366 | 245.183 |
|  | 4:06 | -30.110 | -196.514 | 136.294 |
|  | 5:30 | -117.936 | -315.748 | 79.876 |
| **Flow CFB** | | | | |
| None (Day 1) | 0:36 | -39.732 | -140.027 | 60.562 |
|  | 1:12 | -30.269 | -128.788 | 68.250 |
|  | 2:06 | -97.000 | -197.338 | 3.338 |
|  | 4:06 | -17.574 | -117.860 | 82.711 |
|  | 5:30 | -60.740 | -162.933 | 41.454 |
| Placebo (Day 84) | 0:36 | -21.711 | -186.695 | 143.272 |
|  | 1:12 | **-175.429** | **-341.464** | **-9.393** |
|  | 2:06 | 10.254 | -156.050 | 176.558 |
|  | 4:06 | -155.759 | -322.076 | 10.558 |
|  | 5:30 | **-191.832** | **-374.787** | **-8.876** |

**Table S2** LSMs CFB with 95% CI of total flow and CFB flow during PLM on study Day 1 and 84. Statistically significant changes (p<0.05) from pre-MMTT values bolded. Abbreviations: CFB = change from baseline; CI = confidence interval; LSM = least squares means; MMTT = mixed meal tolerance test; PLM = passive leg movement.
