# Supplementary material for: Microvascular effects of a mixed meal tolerance test: a model validation study
Source: Clin Physiol Funct Imaging. 2024 Sep 23;45(1):e12904. doi: 10.1111/cpf.12904 (PMC11650408; doi:10.1111/cpf.12904)
Supplement: Supplementary file 4 — Supporting information. [file CPF-45-0-s001.docx]

Supplemental figure captions

Figure 1 Overview of employed imaging techniques and timepoints of assessments pre- and post MMTT administration. Timepoints with statistically significant difference from baseline marked with *. Abbreviations: LSCI = laser speckle contrast imaging; LTH = local thermal hyperaemia; min = minutes; MMTT = mixed meal tolerance test; NO = nitric oxide; PLM = passive leg movement; PORH = post occlusive reactive hyperaemia; SDFM = sidestream dark field microscopy.
